# Dynamic Endothelial Stalk Cell–Matrix Interactions Regulate Angiogenic Sprout Diameter

**DOI:** 10.3389/fbioe.2021.620128

**Published:** 2021-03-19

**Authors:** William Y. Wang, Evan H. Jarman, Daphne Lin, Brendon M. Baker

**Affiliations:** Department of Biomedical Engineering, University of Michigan, Ann Arbor, MI, United States

**Keywords:** angiogenesis, endothelial cell, extracellular matrix, cell migration, microfluidic “lab-on-a-chip,”, proteolysis, cytoskeletal forces, cell proliferation

## Abstract

Angiogenesis is a complex, multicellular process that involves bidirectional interactions between extracellular matrix (ECM) and collectively invading endothelial cell (EC) sprouts that extend the microvasculature during development, wound healing, and disease processes. While many aspects of angiogenesis have been well studied, the relationship between endothelial sprout morphology and subsequent neovessel function remains relatively unknown. Here, we investigated how various soluble and physical matrix cues that regulate endothelial sprouting speed and proliferation correspond to changes in sprout morphology, namely, sprout stalk diameter. We found that sprout stalk cells utilize a combination of cytoskeletal forces and proteolysis to physically compact and degrade the surrounding matrix, thus creating sufficient space in three-dimensional (3D) ECM for lateral expansion. As increasing sprout diameter precedes lumenization to generate perfusable neovessels, this work highlights how dynamic endothelial stalk cell–ECM interactions promote the generation of functional neovessels during sprouting angiogenesis to provide insight into the design of vascularized, implantable biomaterials.

## Introduction

Vasculature is a hierarchical network of blood vessels with a wide range of diameters spanning the micron (capillaries) to centimeter (aorta) length scales. A major focus in the fields of biomaterials and tissue engineering has been placed on engineering microvasculature (<50 μm diameter range), as without functional microvasculature, implantable cell-dense tissue constructs are limited in size (<200 μm thickness) due to insufficient gas/nutrient/waste exchange (Huxley and Rumbaut, 2000; Novosel et al., 2011; Kinstlinger and Miller, 2016). One such strategy to resolve the vascularization challenge lies in the design of biomaterials that promote host angiogenesis, the extension of microvasculature from the host’s preexisting vessels into the implant, thereby ensuring circulation between implant and host. Observations consistent across a wide range of *in vivo* and *in vitro* models of angiogenesis have established several key steps including (1) chemokine gradients promoting endothelial tip cell formation and directed invasion into the extracellular matrix (ECM); (2) collective migration of leading tip cells and ensuing stalk cells; and (3) proliferation and expansion of stalk cells into lumenized, fluid-bearing neovessels (Francavilla et al., 2009; Potente et al., 2011). Each of these steps is regulated by both biochemical and physical microenvironmental cues provided by the surrounding ECM, the three-dimensional (3D) fibrous, collagenous meshwork through which endothelial sprouts navigate (Crosby and Zoldan, 2019).

While many aspects of angiogenesis have been studied, continued efforts are required to better understand the relationship between endothelial cell (EC) invasion morphology and subsequent neovessel function. Recent work by our group and others has established how cell intrinsic features (contractility and cell–cell adhesions), soluble factors (chemoattractants and mitogens), and matrix properties (matrix density, degradability, and stiffness) regulate the multicellularity and connectivity of invading EC sprouts, which are critical factors to the ultimate function of neovessels (e.g., perfusability and permeability) (Trappmann et al., 2017; Yoon et al., 2019; Wang et al., 2020). Beyond sprout multicellularity, prior work has also established that the speed of sprout invasion impacts their resulting geometry, where sprout length has been observed to anti-correlate with diameter (Wood et al., 2012). While sprout invasion speed and resulting length are critical determinants of the thickness of an implant that can be vascularized before hypoxia takes hold, sprout diameter is equally important: insufficiently sized sprout diameters may prevent subsequent lumenization required for generating fluid-bearing neovessels (Van Hinsbergh and Koolwijk, 2008). While matrix proteolysis has been the central focus on how invading EC sprouts may create space in 3D ECM to migrate and lumenize (Chun et al., 2004; Van Hinsbergh and Koolwijk, 2008), studies from single-cell encapsulation of mesenchymal stem cells, fibroblasts, and cancer cells have elucidated other means of 3D ECM reorganization that dictate cell shape and subsequent function such as proliferation, migration, and differentiation (Baker et al., 2015; Chaudhuri et al., 2016; Yamada and Sixt, 2019). Continued investigation is required to understand structure–function relationships in multicellular contexts such as collectively invading EC sprouts during angiogenesis.

Extracellular matrix properties regulate the morphologies, migratory modes, and cellular machineries used by cells to spread and migrate in confining 3D microenvironments. Within sufficiently porous and pliable environments, cells can navigate through a confining ECM meshwork by squeezing through pores or utilizing cell forces to physically deform the 3D space to move the cell body forward (Denais et al., 2016; Wisdom et al., 2018; Wang et al., 2019; Ilina et al., 2020). Cell protrusions driven by cooperative cytoskeletal filaments, primarily coordinated microtubule cores and actin-rich tips, engage adhesive ECM binding sites and apply pushing or pulling forces that reorganize the surrounding matrix (Rhee et al., 2007; Baker et al., 2015; Hall et al., 2016; Dogterom and Koenderink, 2019; Shakiba et al., 2020). However, in ECM with smaller pores relative to the migrating cell unit, proteolytic degradation of the matrix is required to create sufficient 3D space into which cells can migrate (Friedl and Wolf, 2009; Wolf and Friedl, 2011). While the majority of these studies have been focused on single cells, recent work has shown that endothelial tip cells apply actomyosin-driven contractile forces to deform the ECM and that tip cell forces generate local collagen fibril alignment to help guide their continued invasion *via* contact guidance (Du et al., 2016; Yoon et al., 2019; Vaeyens et al., 2020). However, how trailing stalk cells of an invading sprout apply forces and remodel the ECM to provide the required space to increase sprout diameter and enable lumenization remains unknown.

In this work, we utilized a multiplexed angiogenesis-on-a-chip platform to examine how various soluble and physical microenvironmental cues regulate endothelial sprout morphology, namely, sprout stalk diameter. We identified soluble cues that modulate sprouting speed and proliferation to be anti- and positively correlated with sprout diameter, respectively. Interestingly, modulating sprouting speed with collagen matrix density resulted in a positive correlation between speed and diameter. As EC sprouts require space in 3D ECM to increase in stalk diameter, we examined how both biochemical remodeling *via* proteolytic matrix degradation and physical pushing forces driven by actomyosin and microtubules compact the ECM surrounding sprout stalk cells to regulate sprout morphology. Overall, this work expands our understanding of how endothelial stalk cells (in addition to previously established tip cells) within angiogenic sprouts dynamically interact with the surrounding ECM to control the morphology and eventual functionality of neovessels formed *via* angiogenesis.

## Materials and Methods

### Reagents

All reagents were purchased from Sigma-Aldrich and used as received, unless otherwise stated.

### Microfluidic Device Fabrication

**FIGURE 1:**
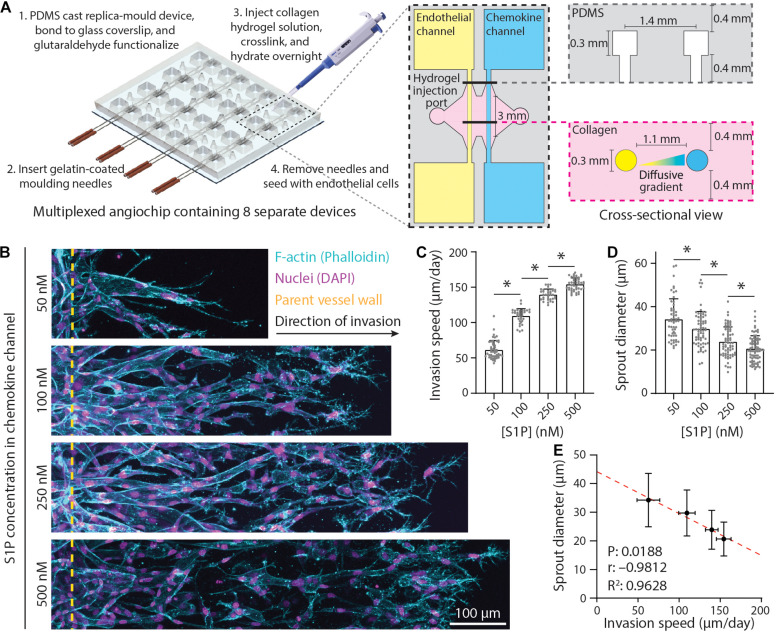
Endothelial sprout invasion speed is anti-correlated with sprout diameter. **(A)** Schematic overview of multiplexed angiogenesis-on-a-chip platform. Polydimethylsiloxane (PDMS) replica casts are generated from 3D printed molds and are composed of a 2 × 4 array of single devices. These PDMS molds are bonded to glass coverslips and functionalized with glutaraldehyde (Step 1). Gelatin-coated needles are then inserted into the device (Step 2). Type 1 collagen hydrogel precursor solution is injected into each device, allowed to cross-link around the needles, and hydrated overnight (Step 3). Needle removal generates 3D channels fully embedded within user-defined hydrogel (Step 4). Each device is composed of two parallel channels: (1) an endothelial channel seeded with endothelial cells (ECs) to serve as the parent vessel from which angiogenic sprouting is induced and (2) a chemokine channel to which pro-angiogenic factors [e.g., sphingosine 1-phosphate (S1P)] are added to generate diffusive gradients that promote EC activation and invasion across a user-defined 3D extracellular matrix (e.g., collagen hydrogel). **(B)** Representative images (max intensity projections) of invading ECs in response to varying S1P. All conditions were cultured for 5 days with indicated S1P dose in endothelial cell growth medium 2 [EGM2; supplemented with 25 ng ml^–1^ phorbol 12-myristate 13-acetate (PMA)] added to the chemokine channel within 3 mg ml^–1^ collagen hydrogels. *F*-actin (cyan), nuclei (magenta), and yellow dashed lines indicate parent vessel edge. **(C,D)** Quantifications of invasion speed and sprout diameter as a function of S1P. For invasion speed: *n* ≥ 36 per condition and for sprout diameter: *n* ≥ 55 per condition. **(E)** Relationship between invasion speed and sprout diameter, with red dashed line indicating a linear regression and statistical analysis performed by Pearson’s correlation. Sample size for each mean was taken from panels **(C,D)**. All data presented as mean ± SD; ^∗^indicates a statistically significant comparison with *P* < 0.05 (one-way analysis of variance).

The 3D printed molds were designed in AutoCAD and printed *via* stereolithography by Protolabs (Maple Plain, MN, United States). Polydimethylsiloxane (PDMS, 1:10 cross-linker:base ratio) devices were replica casted from 3D printed molds, cleaned with isopropyl alcohol and ethanol, and bonded to glass coverslips activated by a plasma etcher. Devices were treated with 0.01% (w/v) poly-L-lysine and 0.5% (w/v) L-glutaraldehyde sequentially for 1 h each (with Milli-Q rinses in between) to promote ECM attachment to the PDMS housing, thus preventing potential hydrogel compaction from cell-generated forces. To generate patent microchannels, 300 μm stainless steel acupuncture needles (Lhasa OMS, Weymouth, MA, United States) were dip-coated with 1% (w/v) gelatin to enable eventual hydrogel release, inserted into each device and sterilized by UV ozone. Hydrogel precursor solution was then injected into each device and polymerized around each set of needles. Hydrogels were hydrated in Endothelial cell growth medium-2 (EGM2) containing 50 mM glycine [to quench unreacted glutaraldehyde (GA)] and incubated at 37°C overnight to dissolve the gelatin layer. Needles were subsequently removed, yielding 3D microchannels fully embedded within a collagen hydrogel positioned 400 μm away from PDMS and glass boundaries (Figure 1A).

### Collagen Hydrogel Formulation

Type I rat tail collagen hydrogels (Corning, Corning, NY, United States) were prepared on ice with a reconstitution buffer (10 mM HEPES, 0.035% w/v sodium bicarbonate, 1 × M199), titrated to a pH of 7.6 with 1 M NaOH, and brought to a final concentration of 2, 3, or 6 mg ml^–1^ collagen. Collagen hydrogels were cross-linked for 30 min at 37°C. All hydrogels were hydrated in EGM2 media after cross-linking. Fluorescently labeled collagen was prepared as in Doyle (2016) and incorporated at 2 wt.% of the total collagen content (1:50 dilution in unlabeled collagen).

### Device Cell Seeding and Culture

Human umbilical vein ECs (HUVECs; Lonza, Switzerland) were cultured in endothelial growth media (EGM2; Lonza). HUVECs were passaged upon achieving confluency at a 1:4 ratio and used in studies from passages 4–9. A 20 μl solution of suspended HUVECs was added to one reservoir of the endothelial channel and inverted for 30 min to allow cell attachment to the top half of the channel, followed by a second seeding with the device upright for 30 min to allow cell attachment to the bottom half of the channel. HUVEC solution density was varied with collagen density as attachment efficiency was dependent on collagen density (Wang et al., 2020). HUVEC seeding densities were determined experimentally to achieve parent vessels with consistent cell densities across each hydrogel formulation (1.5 M ml^–1^ for 2 mg ml^–1^, 2 M ml^–1^ for 3 mg ml^–1^, and 5 M ml^–1^ for 6 mg ml^–1^). HUVECs reached confluency and self-assembled into stable parent vessels over 24 h. Media and chemokines were refreshed every 24 h, and devices were cultured with continual reciprocating flow utilizing hydrostatic pressure-driven flow on a seesaw rocker plate at 0.33 Hz. For pharmacological studies, 25 μM blebbistatin (Santa Cruz Biotechnology, Dallas, TX, United States), 50 ng ml^–1^ nocodazole, and 1 μM marimastat were added to both endothelial and chemokine channel media and refreshed every 24 h.

### Fluorescent Staining

Samples were fixed with 4% paraformaldehyde and permeabilized with a phosphate-buffered saline (PBS) solution containing Triton X-100 (5% v/v), sucrose (10% w/v), and magnesium chloride (0.6% w/v) for 1 h each at room temperature. AlexaFluor 488 phalloidin (diluted 1:500; Life Technologies, Carlsbad, CA, United States) was utilized to visualize *F*-actin. In addition, 4′,6-diamidino-2-phenylindole (DAPI; 1 μg ml^–1^) was utilized to visualize cell nuclei. For proliferation studies, 5-ethynyl-2′-deoxyuridine (EdU) was applied for the final 24 h prior to fixation of each study. EdU fluorescent labeling was performed following the manufacturer’s protocol (ClickIT EdU, Life Technologies). DyLight 649 labeled Ulex Europaeus Agglutinin-1 (UEA, 1:200, Vector Labs, Burlingame, CA, United States) was utilized to visualize EC morphology in samples stained with EdU due to the incompatibility of EdU ClickIT chemistry with phalloidin staining. To visualize α-tubulin, samples were sequentially blocked in bovine serum albumin (0.3% w/v), incubated with primary mouse monoclonal anti-α-tubulin (1:200, Invitrogen), and incubated with secondary AlexaFluor 647 goat anti-mouse IgG (H+L) (1:1,000; Life Technologies) each for 8 h at room temperature. To visualize collagen degradation, collagen hybridizing peptide (5 μM; 3 Helix, Salt Lake City, Utah) was heated to 80°C for 5 min, then chilled in an ice-water bath for 30 s, and added to samples for 8 h.

### Microscopy and Image Analysis

Fluorescent images were captured on a Zeiss LSM800 confocal microscope. Time-lapse imaging was performed on sprouts after 2 days of culture in an environmentally controlled chamber (37°C, 5% CO_2_, and 100% humidity) with images acquired every 20 min over 8 h. For time-lapse imaging with 24-h frame intervals, devices were cultured in the incubator and transported to the microscope stage for daily imaging in a custom stage holder such that specific regions of interest were maintained over the 5-day imaging period. EC density and proliferation (EdU-positive nuclei) within sprouts were quantified by counting DAPI and EdU-positive cell nuclei. Invasion depth was quantified as the distance from the parent vessel edge to each sprout’s tip cell and measured manually in FIJI at 100 μm intervals along the parent vessel. Sprout diameter measurements were taken orthogonal to the long axis of the sprout, 30–50 μm away from the parent vessel edge. Collagen compaction analyses were performed by acquiring intensity profiles along the sprout stalk region orthogonal to the long axis of the sprout, 30–50 μm away from the parent vessel edge (i.e., identical region to sprout diameter measurements). Given differences in fluorescent intensity as a function of initial collagen hydrogel density, images of acellular regions were used to determine baseline collagen intensity to normalize measurements following remodeling. The intensity fold change of the compacted collagen area (sprout periphery) compared to the baseline collagen intensity was utilized as a relative measure of collagen compaction.

### Statistics

Statistical significance was determined by one-way analysis of variance (ANOVA), two-sided Student’s *t*-test, or Pearson’s correlation where appropriate, with significance indicated by *P* < 0.05. Pearson’s correlation was performed on sample mean values for each group without accounting for total sample size; a strong correlation was defined as *R*^2^ > 0.7. Sample size is indicated within corresponding figure legends, and all data are presented as mean ± standard deviation.

## Results

### Endothelial Cell Migration Speed and Proliferation Influence Sprout Diameter

To investigate how ECM remodeling events influence angiogenic sprout morphology, we implemented a recently established multiplexed angiogenesis-on-a-chip platform that affords improved experimental throughput to explore a wide parameter space of soluble and physical cues (Figure 1A; Wang et al., 2020). This microfluidic-based device has been shown to recapitulate 3D EC sprouting morphogenesis from the stable, quiescent endothelium of a parent vessel (Nguyen et al., 2013; Trappmann et al., 2017; Yoon et al., 2019; Wang et al., 2020). The parent vessels modeled in this work possess a diameter (300 μm) that lies near the upper end of values previously described for arterioles but lack support cells such as vascular smooth muscle cells or pericytes; although these additional cell types can be included with this approach, we focused here on how microenvironmental cues affect ECs in the absence of confounding cross talk between cocultured cell types (Kinstlinger and Miller, 2016; Alimperti et al., 2017; Traore and George, 2017). To induce EC invasion into 3D ECM, we introduced an established EC chemoattractant, sphingosine 1-phosphate (S1P), to the adjacent chemokine channel to produce a diffusive gradient that drives EC activation and directional 3D invasion (Paik et al., 2001; Nguyen et al., 2013; Wang et al., 2020). As prior work has demonstrated that sprout invasion speed is anti-correlated with sprout diameter (Wood et al., 2012), we first investigated the effect of S1P on sprout morphology, as our previous studies indicate an S1P dose-dependent increase in invasion speed (Wang et al., 2020). Indeed, increasing S1P resulted in higher sprout invasion speeds (Figures 1B,C). As the diameter along the length of a sprout was variable, we measured sprout diameters toward the sprout stalk region (30–50 μm away from the parent vessel edge and orthogonal to the long axis of each sprout). With increasing S1P and greater invasion speeds, sprout diameters decreased, producing a significant and strong negative correlation between invasion speed and sprout diameter (Figures 1D,E). In sum, this result within a distinct model system supports findings from prior studies (Wood et al., 2012).

**FIGURE 2:**
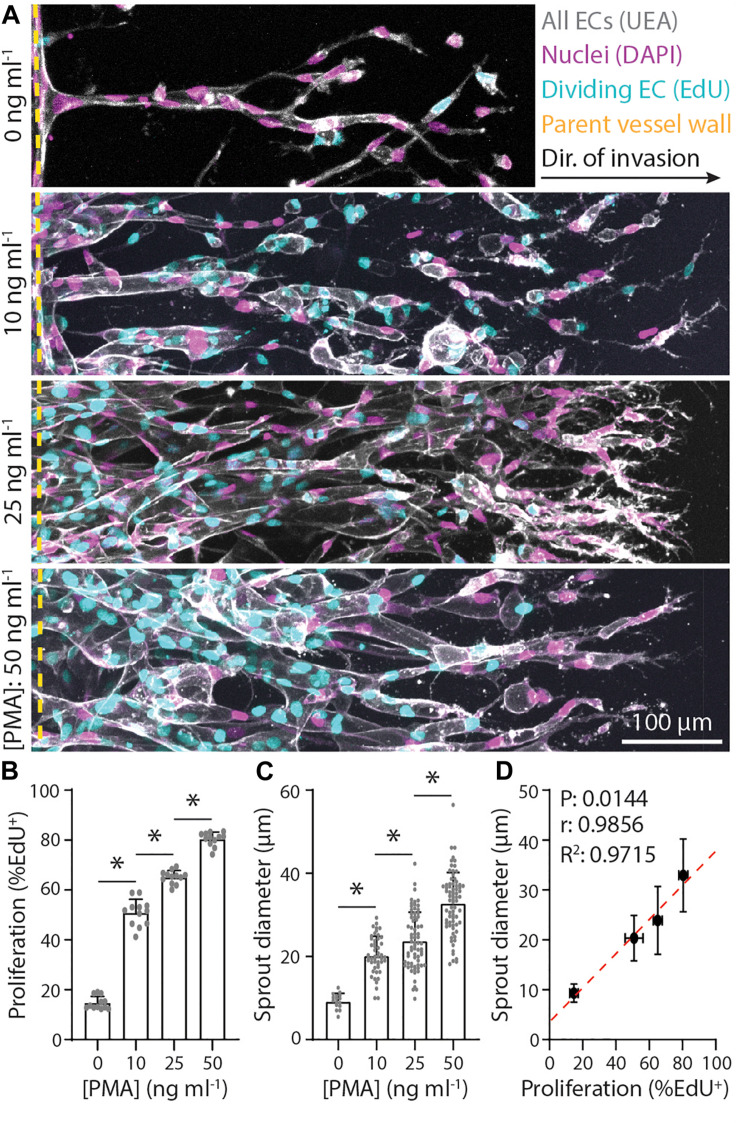
Endothelial stalk cell proliferation is positively correlated with sprout diameter. **(A)** Representative images (max intensity projections) of invading endothelial cells (ECs) in response to varying phorbol 12-myristate 13-acetate (PMA). All conditions were cultured for 5 days with 250 nM sphingosine 1-phosphate (S1P) in endothelial cell growth medium 2 (EGM2; supplemented with indicated PMA) added to the chemokine channel within 3 mg ml^–1^ collagen hydrogels. Ulex Europaeus Agglutinin-1 (UEA; white), nuclei (magenta), 5-ethynyl-2′-deoxyuridine (EdU; cyan), and yellow dashed lines indicate parent vessel edge. **(B,C)** Quantifications of proliferation and sprout diameter as a function of PMA. For proliferation: *n* ≥ 12 per condition and for sprout diameter: *n* ≥ 16 per condition. **(D)** Relationship between proliferation and sprout diameter, with red dashed line indicating a linear regression and statistical analysis performed by Pearson’s correlation. Sample size for each mean is identical to those of panels **(B,C)**. All data presented as mean ± SD; *indicates a statistically significant comparison with *P* < 0.05 (one-way ANOVA).

In addition to chemoattractant-mediated directional EC invasion, another key requirement of angiogenesis is sufficient cell proliferation, which occurs predominantly within stalk cells of an invading angiogenic sprout (Gerhardt et al., 2003). To investigate the relationship between proliferation and sprout morphology, we supplemented EGM2 media with varying concentrations of phorbol 12-myristate 13-acetate (PMA), a well-established pro-angiogenic factor and potent activator of protein kinase C (PKC) upstream of cell proliferation (Cross et al., 2010; Nguyen et al., 2013; Osaki et al., 2015; Wang et al., 2020). To assess EC proliferation, we utilized an EdU assay that labels proliferating cell nuclei, while all cells were labeled with DAPI and UEA. Indeed, EC proliferation proved dose-dependent with PMA, where increasing PMA resulted in stepwise increases in proliferation rates (the ratio of EdU^+^ cells to all cells labeled with DAPI and UEA) (Figures 2A,B). Sprout diameter was also found to be dose-dependent with PMA, where increasing PMA led to larger diameters, yielding a significant and strong positive correlation between EC proliferation and sprout diameter (Figures 2C,D). Taken together, soluble factors that drive cell invasion (i.e., S1P) and proliferation (i.e., PMA) differentially regulate resulting sprout morphology (i.e., diameter). Our previous work demonstrates a critical balance between these two fundamental cellular functions in maintaining cell–cell adhesion during collective sprout invasion (Wang et al., 2020). These findings suggest that a similar balance is required for invading ECs to generate sprout diameters in the <50 μm diameter range reported for microvasculature *in vivo* (Aird, 2005; Kinstlinger and Miller, 2016; Traore and George, 2017).

### Matrix Density Regulates Sprouting Speed and Diameter

**FIGURE 3:**
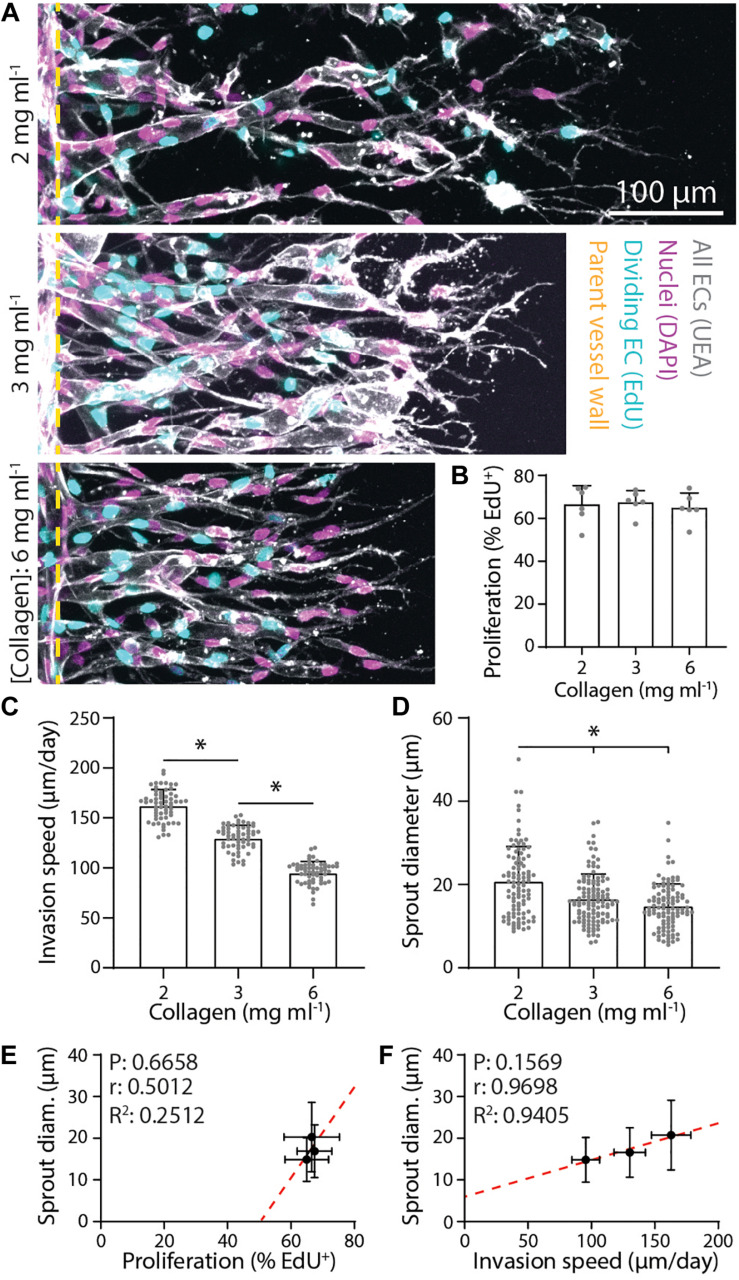
Denser matrix that slows sprout invasion leads to smaller sprout diameters. **(A)** Representative images (max intensity projections) of invading endothelial cells (ECs) in response to varying collagen density. All conditions were cultured for 3 days with 250 nM sphingosine 1-phosphate (S1P) in endothelial cell growth medium 2 [EGM2; supplemented with 25 ng ml^–1^ phorbol 12-myristate 13-acetate (PMA)] added to the chemokine channel within collagen hydrogels of the indicated density. Nuclei (magenta), 5-ethynyl-2′-deoxyuridine (EdU; cyan), Ulex Europaeus Agglutinin-1 (UEA; white), and yellow dashed lines indicate parent vessel edge. **(B–D)** Quantifications of proliferation, invasion speed, and sprout diameter as a function of collagen hydrogel density. For proliferation: *n* ≥ 6 per condition, for invasion speed: *n* ≥ 60 per condition, and for sprout diameter: *n* ≥ 100 per condition. **(E,F)** Relationships between proliferation and sprout diameter **(E)** and invasion speed and sprout diameter **(F)**, with red dashed lines indicating a linear regression and statistical analyses performed by Pearson’s correlation. Sample size for each mean is identical to those of panels **(B–D)**. All data presented as mean ± SD; *indicates a statistically significant comparison with *P* < 0.05 (one-way ANOVA).

Beyond soluble biochemical factors, physical properties of ECM have also been shown to modulate angiogenesis (Crosby and Zoldan, 2019; Wang et al., 2020). We next investigated the influence of matrix density on sprout morphology (all previous studies were performed in 3 mg ml^–1^ collagen) under constant levels of S1P (250 nM) and PMA (25 ng ml^–1^). With increasing collagen density, we observed no change in proliferation rates (perhaps due to PMA’s potent effect on proliferation) but stepwise decreases in invasion speeds (Figures 3A–C). Despite the reduction in invasion speeds, sprout diameter surprisingly decreased with increasing matrix density (Figure 3D). We observed a weak positive correlation between proliferation and sprout diameter and a strong positive correlation between invasion speed and sprout diameter (Figures 3E,F). We note that none of these correlations proved significant likely due to the low number of groups in the analysis. Thus, while diminishing the S1P chemoattractive gradient and increasing collagen density both act to slow sprout invasion, decreased invasion speeds do not consistently correlate with thicker sprouts. Modulating S1P in 3 mg ml^–1^ collagen yields a significant and strong negative correlation between speed and diameter while modulating matrix density results in a strong positive correlation.

### Dynamic Sprout–Extracellular Matrix Interactions Regulate Sprout Diameter

**FIGURE 4:**
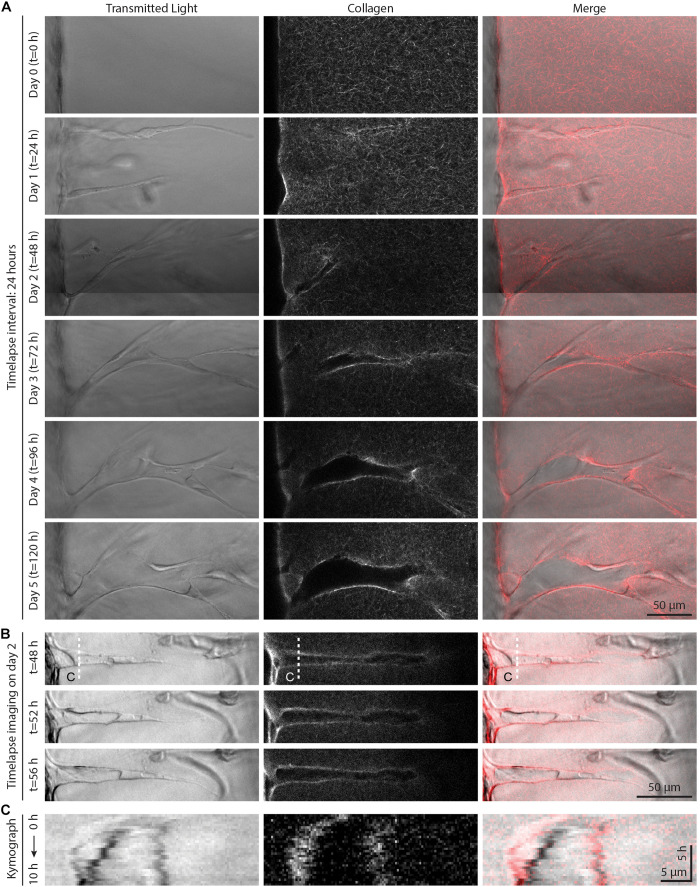
Live time-lapse imaging reveals dynamic sprout and extracellular matrix (ECM) interactions. **(A)** Representative time course images (individual z-slices) of an invading sprout over 5 days with a frame interval of 24 h. Sprouts were cultured in 250 nM sphingosine 1-phosphate (S1P) and 50 ng ml^–1^ phorbol 12-myristate 13-acetate (PMA) within 3 mg ml^–1^ collagen hydrogel. **(B)** Representative time course images (individual z-slices) of an invading sprout over 8 h with a frame interval of 20 min (for full time-lapse series, see Supplementary Video 1). Sprouts were cultured in 250 nM S1P and 50 ng ml^–1^ PMA within 3 mg ml^–1^ collagen hydrogel, and imaging was initiated after 2 days in culture. **(C)** Kymographs taken along white dashed line indicated in panel **(B)**.

To further investigate these seemingly contradictory invasion speed vs. sprout diameter relationships, we employed live time-lapse imaging with fluorescently labeled collagen to capture dynamic cell–ECM interactions over the course of EC sprouting through 3D ECM. As angiogenic sprouting occurs over several days, we first performed live cell imaging over 24 h imaging intervals to examine long-term changes (Figure 4A). Imaging the same region over 5 days, we observed the invasion of an endothelial tip cell at day 1 with no appreciable changes to the surrounding ECM structure or density. By day 2, tip cells continued invading and led ensuing stalk cells, with localized collagen degradation evident at the location of the sprout stalk. Over days 3–5, sprouts continued to elongate as well as expand laterally, increasing in diameter. Interestingly, the area devoid of collagen continued to grow with expansion of the sprout, with marked increases of collagen fluorescence intensity at the sprout periphery. This observation suggests collagen is not only proteolytically degraded but also physically compacted by stalk cells to accommodate the expanding sprout. We next employed time-lapse imaging at shorter frame intervals (every 20 min over 8 h) on day 2 of culture to capture more transient cell–ECM interactions (Figures 4B,C and Supplementary Video 1). The dynamics of sprout morphology (fluctuations in sprout diameter) mirrored that of the surrounding collagen matrix, with the compacted peripheral zone of collagen moving in tandem with the expanding diameter of the sprout (best viewed in Supplementary Video 1). Live cell imaging over these two different timescales suggests that over shorter durations (<1 day), invading sprouts appear to dynamically displace the surrounding collagen through active cellular shape changes and resulting forces. Over longer timescales (>1 day), sprouts can degradatively remodel the collagen to create space for the growing sprout.

**FIGURE 5:**
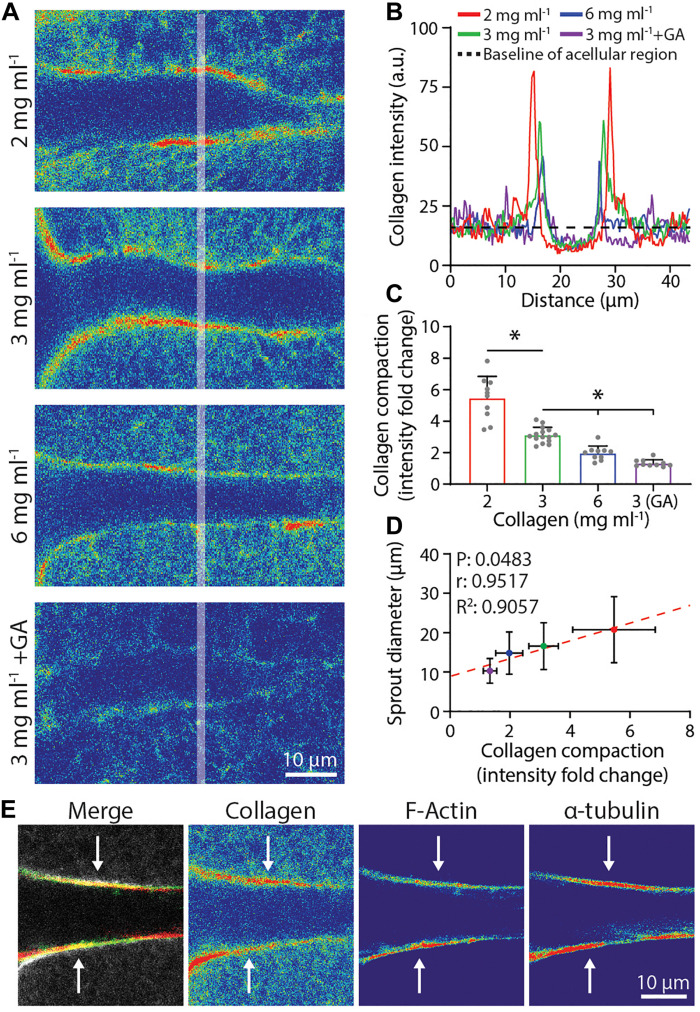
Degree of collagen compaction adjacent to sprout stalk cells positively correlates with sprout diameter. **(A)** Representative images (individual z-slices) of fluorescently labeled collagen (shown as intensity heat map) surrounding sprout stalk cells in collagen matrices of varying density and stiffness. Sprouts were cultured for 3 days in 250 nM sphingosine 1-phosphate (S1P) and 50 ng ml^–1^ phorbol 12-myristate 13-acetate (PMA) within collagen matrices of indicated density or with glutaraldehyde (GA) cross-linking. **(B)** Representative fluorescent collagen intensity profiles acquired along shaded white line indicated in panel **(A)**. 2 mg ml^–1^ (red line), 3 mg ml^–1^ (green line), 6 mg ml^–1^ (blue line), and 3 mg ml^–1^ with GA cross-linking (purple line) and average intensity of non-compacted, acellular areas (dashed black line). Intensity values of the non-compacted, acellular collagen regions were normalized to each other in panels **(A,B)** to compare collagen intensity fold change of compacted regions. **(C,D)** Quantification of collagen compaction and relationship between collagen compaction and sprout diameter, with red dashed line indicating a linear regression and statistical analysis performed by Pearson’s correlation. For collagen compaction: *n* ≥ 10 per condition. For each mean in panel **(D)**, *n* ≥ 10 for collagen compaction and *n* ≥ 70 for sprout diameter. **(E)** Representative images (individual *z*-slices) of sprout stalk region stained for *F*-actin (green) and α-tubulin (red) within fluorescently labeled collagen (gray). Individual channels visualized as intensity heat maps. All data presented as mean ± SD; *indicates a statistically significant comparison with *P* < 0.05 (one-way ANOVA).

Given that both physical compaction and degradative remodeling would be significantly influenced by matrix density, we next examined sprout stalk-driven collagen compaction as a function of collagen density. To measure a relative degree of collagen compaction, sprouts were allowed to invade in fluorescent collagen and line intensity profiles orthogonal to the long axis of each sprout 30–50 μm away from the parent vessel edge were acquired by confocal imaging (shaded lines in Figure 5A). An intensity fold change was determined by normalizing fluorescence intensity to a baseline collagen intensity from surrounding acellular regions (Figure 5B). Collagen compaction diminished with increasing collagen density, suggesting that with higher collagen density, expansive stalk cell forces are no longer sufficient to compact the surrounding matrix, thereby restricting lateral expansion of growing sprouts (Figure 5C). Supporting this, we noted a significant and strong positive correlation between the degree of collagen compaction and sprout diameter (Figure 5D). Additionally, GA treatment of collagen hydrogels, which introduces non-cell-degradable cross-links and increases collagen gel stiffness, also reduced the degree of collagen compaction and sprout diameter (Figures 5A–D). As cytoskeletal proteins are responsible for cellular shape changes associated with forces applied to the ECM, we co-stained sprouts for *F*-actin and α-tubulin. *F*-actin and α-tubulin were both enriched and co-localized along the periphery of sprout stalks where collagen compaction was also most pronounced, suggesting that actomyosin and microtubules provide the driving forces behind matrix compaction (Figure 5E). Taken together, sprout stalk cells dynamically engage and deform the surrounding ECM and over longer timescales, can permanently remodel the structure to create sufficient space for the growing sprout.

### Actomyosin, Microtubules, and Proteolysis Regulate Sprout Diameter and Extracellular Matrix Compaction

**FIGURE 6:**
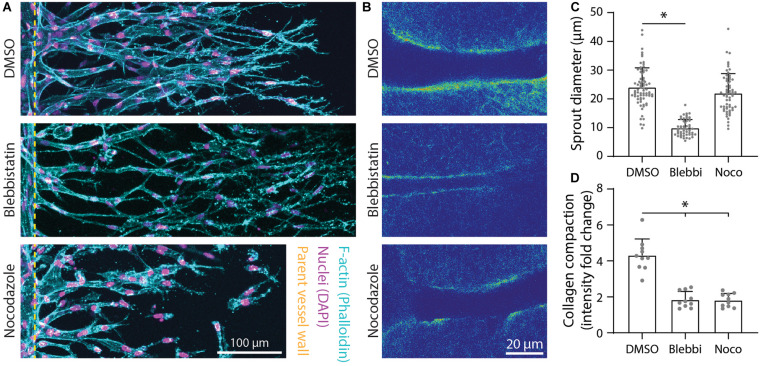
Inhibition of actomyosin activity and microtubule polymerization reduces collagen compaction. **(A)** Representative images (max intensity projections) of dimethylsulfoxide (DMSO) control, 25 μM blebbistatin-, and 50 ng ml^–1^ nocodazole-treated sprouts cultured over 5 days with 250 nM sphingosine 1-phosphate (S1P) and 25 ng ml^–1^ phorbol 12-myristate 13-acetate (PMA) within 3 mg ml^–1^ collagen. **(B)** Representative images (individual z-slices) of fluorescently labeled collagen (intensity heat map) of conditions from panel **(A)**. **(C,D)** Quantifications of sprout diameter and collagen compaction from conditions in panels **(A,B)**. For sprout diameter: *n* ≥ 52 per condition and for collagen compaction: *n* ≥ 10 per condition. All data presented as mean ± SD; *indicates a statistically significant comparison to DMSO control with *P* < 0.05 (two-tailed Student’s *t*-test).

As *F*-actin and α-tubulin appeared to be enriched at areas of collagen compaction at the sprout stalk periphery and are known drivers of cell shape and cell-mediated matrix deformations, we used pharmacologic inhibitors of actomyosin activity and microtubule assembly to test whether they cooperatively regulate sprout diameter and ECM compaction. The addition of 25 μM blebbistatin, a myosin II inhibitor, resulted in decreased sprout diameters with corresponding decreases in collagen compaction (Figures 6A–D). Reducing microtubule assembly with the addition of 50 ng ml^–1^ nocodazole also resulted in decreased collagen compaction but without commensurate decreases in sprout diameter (Figures 6A–D). Live imaging revealed nocodazole-treated stalk cells lose connectivity to tip cells and retract collectively toward the parent vessel (Supplementary Video 2). In retracting and reducing their length, stalk cells condensed, and expanded laterally. Lacking the ability to re-extend, nocodazole-treated sprouts were shorter in length (i.e., invasion depth) and sprout diameters were not significantly different compared to controls. Sprouts treated with podophyllotoxin, which fully prevents microtubule assembly, resulted in disassembly of multicellular sprouts into individual cells (Supplementary Video 3).

**FIGURE 7:**
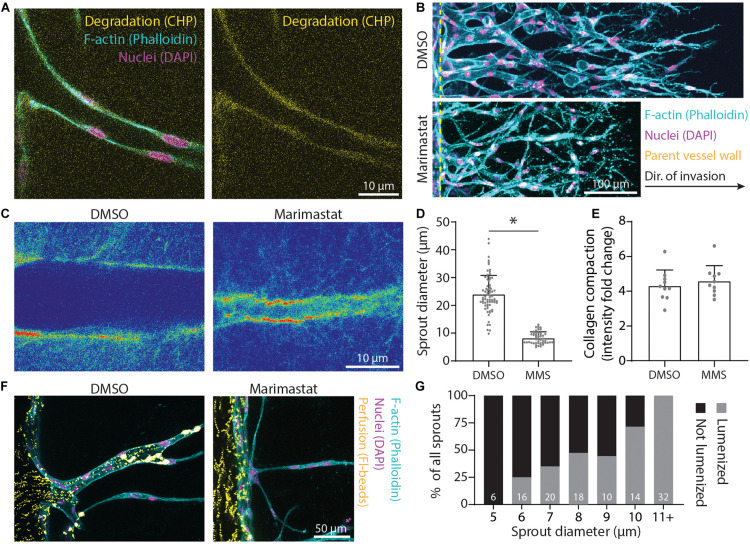
Matrix proteolysis is required for larger sprout diameters that are lumenized. **(A)** Representative image (individual *z*-slices) of collagen degradation along sprout stalk cells. *F*-actin (cyan), nuclei (magenta), collagen hybridization peptide (CHP; yellow). **(B)** Representative images (max intensity projections) of marimastat (MMS) treatment (1 μM) of sprouts cultured over 5 days with 250 nM sphingosine 1-phosphate (S1P) and 25 ng ml^–1^ phorbol 12-myristate 13-acetate (PMA) within 3 mg ml^–1^ collagen. **(C)** Representative images (single z-slice) of fluorescently labeled collagen (intensity heat map) with MMS treatment (1 μM) of sprouts cultured over 3 days with 250 nM S1P and 25 ng ml^–1^ PMA within 3 mg ml^–1^ collagen. **(D,E)** Quantifications of sprout diameter and collagen compaction from MMS treatment (1 μM). For sprout diameter: *n* ≥ 50 per condition and for collagen compaction: *n* ≥ 10 per condition. **(F,G)** Assessment of lumenization as a function of sprout diameter by parent vessel perfusion of 1 μm-diameter microsphere. Sample size for total sprouts analyzed for each group is indicated in bar plot. All data presented as mean ± SD; *indicates a statistically significant comparison with *P* < 0.05 (two-tailed Student’s *t*-test).

In addition to physical reorganization of ECM, EC sprouts also enzymatically degrade the ECM by using matrix metalloproteinases (MMPs) to create space in 3D. Utilizing a collagen hybridization peptide (CHP), which binds to degraded collagen (individual collagen peptides cleaved from tropo-collagen), we found CHP to be enhanced along the periphery of sprout stalks relative to acellular ECM regions (Figure 7A). The addition of 1 μM marimastat, a broad-spectrum MMP inhibitor, over 5 days of culture resulted in decreased sprout diameter but without changes to the degree of collagen compaction (Figures 7B–E). To assess whether sprout diameter impacts subsequent lumenization and perfusability, we perfused 1 μm-diameter fluorescent microspheres through the endothelial channel. We found that all sprouts with a diameter < 6 μm were incapable of supporting microsphere perfusion within the multicellular sprout, while all sprouts with a diameter ≥ 11 μm were lumenized, as evident by the presence of microspheres within sprouts (Figures 7F,G). Sprouts with diameters between 6 and 11 μm displayed a stepwise increase in the percentage of sprouts that were perfused with microspheres with increasing sprout diameter (Figure 7G). Taken together, EC sprouts create space in 3D ECM with a combination of biochemical and physical means to afford increases in sprout diameter and lumenization. Inhibition of actomyosin and microtubules reduced collagen compaction around sprout stalk cells and are critical regulators of maintaining EC invasion morphology as collective, multicellular strands (Figure 6). Reduction of matrix proteolysis decreases sprout diameter; however, persistent actomyosin and microtubule-driven expansive forces were sufficient to physically reorganize ECM and enable the invasion of thin multicellular strands (Figure 7).

## Discussion

The relationship between microenvironmental cues, angiogenic sprout morphology, and subsequent neovessel function remains relatively understudied. Our group recently established that the multicellularity of invading ECs dictates the subsequent function (perfusability and permeability) of formed neovessels (Trappmann et al., 2017; Wang et al., 2020). The work presented here builds upon this structure–function relationship, highlighting how cytoskeletal and proteolytic machinery of sprout stalk cells enzymatically remodel and mechanically compact the surrounding ECM to create sufficient space to laterally expand sprout stalks and enable lumenization. With increased collagen matrix density or stiffening from GA cross-linking, stalk cell-mediated collagen compaction decreased as cell-generated forces were likely insufficient to compact a more mechanically resistant ECM, thus yielding smaller sprout diameters (Figure 3). Additionally, increasing matrix density or stiffening with GA cross-linking led to a stiffer ECM more resistant to proteolysis, thus requiring enhanced MMP activity and/or cell-generated forces to generate open space for growing sprouts. Modulating EC proliferation with PMA, we found sprout diameters increased with increases in stalk cell proliferation (Figure 2); we anticipate cell-generated forces and proteolysis are likely cell density dependent, where increased cell density may lead to locally higher levels of multicellular force generation and higher rates of proteolysis, thus resulting in enhanced ECM degradation and compaction that afford more space for larger sprout diameters. Lastly, cytoskeletal forces and proteolysis remodeled the ECM more drastically over longer timescales (days) (Figure 4); modulating sprouting speed with soluble chemoattractants resulted in faster invasion speeds and decreased sprout diameters, which may spatiotemporally limit physical and proteolytic interactions between stalk cells and adjacent matrix (Figure 1).

Toward the design of pro-angiogenic biomaterials that promote host angiogenesis, this work highlights a critical need for biomaterial cues that promote sprout stalk cell-mediated increases in diameter and subsequent lumenization. While natural materials such as collagen and fibrin hydrogels offer sufficient microporosity and enable cell-mediated remodeling to create space, following implantation, these materials are rapidly resorbed and lose their initial structural integrity (Thomson et al., 2013; Vigen et al., 2014). Thus, a major focus in the design of pro-angiogenic biomaterials has been placed on designing tunable synthetic hydrogels with enhanced specificity over degradative mechanisms and tunability over resorption rates to retain integrity upon implantation (Li et al., 2017). Facilitating sprout-mediated matrix remodeling by modulating synthetic hydrogel degradability could augment sprout diameters *via* proteolysis but at the cost of also enhancing resorption rates. Viscoelastic hydrogels could take advantage of proteolysis-independent methods of space generation *via* cell force-mediated pushing forces, as previous studies have demonstrated plastic deformation of hydrogels with viscoelastic behavior (Chaudhuri et al., 2020; Wei et al., 2020). As increased EC proliferation resulted in enhanced sprout diameters, incorporating matrix cues that additionally enhance proliferation in 3D synthetic hydrogel settings would also be beneficial. While matrix stiffness in 2D settings enhances proliferation, increased stiffness in 3D has been shown to decrease cell proliferation and spreading likely due to the confinement of cells in typically nanoporous synthetic hydrogels and the requirement for significant degradation prior to cell spreading, which appears to be a prerequisite for proliferation of adherent cells (Khetan et al., 2013). As native ECM is primarily composed of fibrillar collagens, recent work from our group has designed composite materials composed of stiff, microscale fibers embedded within soft bulk hydrogels (Matera et al., 2019, 2020). These composite hydrogels containing stiff adhesive structures that promote cell spreading and proliferation could have utility for angiogenesis (Matera et al., 2019, 2020). Lastly, generating sufficient space within nanoporous synthetic hydrogels can be approached with subtractive materials engineering methods rather than a cell-mediated process. Recent techniques in 3D printing vessel conduits, photoablation of microtracks, and microporous annealed particles are capable of generating material porosity at various length scales to enhance vascularization (Griffin et al., 2015; Mirabella et al., 2017; Arakawa et al., 2020). The continued advancement of synthetic biomaterials and careful consideration of dynamic sprout-mediated matrix reorganization will be critical for the future development of pro-angiogenic biomaterial implants.

While much progress has been made on understanding how matrix properties regulate sprouting angiogenesis, only recently have we begun to appreciate the dynamic bidirectional interactions between ECM and collectively invading EC sprouts (van Helvert et al., 2018). Most studies define and characterize the initial matrix state and then measure the subsequent cell response. However, as highlighted by this work, EC sprouts dynamically reorganize and remodel ECM structure and mechanics using cytoskeletal forces and proteolytic activity over extended timescales required for complex morphogenetic processes. To understand how cell-altered ECM iteratively and reciprocally influences cell processes requires advances in characterization techniques that probe cell–matrix interactions across space and time. Here, we employed live time-lapse imaging of fluorescently labeled ECM to characterize matrix compaction mirroring sprout morphology fluctuations over short timescales (minutes) and matrix degradation and compaction corresponding to increases in sprout diameter over longer timescales (days). To more closely examine how cells may dynamically alter the matrix state, future work could integrate recent advances in metabolic labeling of secreted proteins to examine matrix deposition (Loebel et al., 2019), fluorescence resonance energy transfer (FRET)-based protease microgels to assess protease activity (Shin et al., 2018), and techniques such as magnetic bead microrheology to spatially characterize matrix stiffness (Juliar et al., 2018). Coupling these matrix state analysis techniques with cell state measurement techniques such as fluorescent fusion-tagged proteins, FRET-based reporters of forces across cytoskeletal proteins (Grashoff et al., 2010; Ham et al., 2019), and transcription factor activity reporters (Aguado et al., 2015) would provide new insights on the dynamic and bidirectional relationships between cells and surrounding matrix. The continued development and deployment of such techniques will be essential in further elucidating critical aspects of cell–matrix reciprocity during the complex and dynamic process of sprouting angiogenesis.

## Data Availability Statement

The raw data supporting the conclusions of this article will be made available by the authors, without undue reservation.

## Author Contributions

WW and BB designed the experiments. WW, EJ, and DL conducted the experiments and analyzed the data. WW and BB wrote the manuscript. All authors reviewed the manuscript.

## Conflict of Interest

The authors declare that the research was conducted in the absence of any commercial or financial relationships that could be construed as a potential conflict of interest.

## Supplementary Material

Supplementary Video 1 Time-lapse imaging of sprout-ECM dynamics cultured in 250 nM S1P and 50 ng ml^–1^ PMA within 3 mg ml^–1^ fluorescently labeled collagen hydrogel. Time-lapse imaging was initiated after 48 h of sprouting. Transmitted light (top), fluorescent collagen (middle), and merge (bottom).

Supplementary Video 2 Time-lapse imaging of nocodazole treated sprouts. Sprouts were cultured in 250 nM S1P and 25 ng ml^–1^ PMA within 3 mg ml^–1^ collagen hydrogel. 50 ng ml^–1^ nocodazole was first administered to sprouts along with the initiation of time-lapse imaging after 48 h of sprouting.

Supplementary Video 3 Time-lapse imaging of podophyllotoxin treated sprouts. Sprouts were cultured in 250 nM S1P and 25 ng ml^–1^ PMA within 3 mg ml^–1^ collagen hydrogel. 100 nM podophyllotoxin was first administered to sprouts along with the initiation of time-lapse imaging after 48 h of sprouting.

## References

[B1] Aguado B. A. Wu J. J. Azarin S. M. Nanavati D. Rao S. S. Bushnell G. G. (2015). Secretome identification of immune cell factors mediating metastatic cell homing. *Sci. Rep.* 5:17566. 10.1038/srep17566 26634905 PMC4669442

[B2] Aird W. C. (2005). Spatial and temporal dynamics of the endothelium. *J. Thromb. Haemost.* 3 1392–1406. 10.1111/j.1538-7836.2005.01328.x 15892866

[B3] Alimperti S. Mirabella T. Bajaj V. Polacheck W. Pirone D. M. (2017). Three-dimensional biomimetic vascular model reveals a RhoA, Rac1, and N -cadherin balance in mural cell–endothelial cell-regulated barrier function. *Proc. Natl. Acad. Sci. U.S.A.* 114 8758–8763. 10.1073/pnas.1618333114 28765370 PMC5565405

[B4] Arakawa C. Gunnarsson C. Howard C. Bernabeu M. Phong K. Yang E. (2020). Biophysical and biomolecular interactions of malaria-infected erythrocytes in engineered human capillaries. *Sci. Adv.* 6:eaay7243. 10.1126/sciadv.aay7243 32010773 PMC6968943

[B5] Baker B. M. Trappmann B. Wang W. Y. Sakar M. S. Kim I. L. Shenoy V. B. (2015). Cell-mediated fibre recruitment drives extracellular matrix mechanosensing in engineered fibrillar microenvironments. *Nat. Mater.* 14 1262–1268. 10.1038/nmat4444 26461445 PMC4654682

[B6] Chaudhuri O. Cooper-White J. Janmey P. A. Mooney D. J. Shenoy V. B. (2020). Effects of extracellular matrix viscoelasticity on cellular behaviour. *Nature* 584 535–546. 10.1038/s41586-020-2612-2 32848221 PMC7676152

[B7] Chaudhuri O. Gu L. Klumpers D. Darnell M. Bencherif S. A. Weaver J. C. (2016). Hydrogels with tunable stress relaxation regulate stem cell fate and activity. *Nat. Mater.* 15 326–334. 10.1038/nmat4489 26618884 PMC4767627

[B8] Chun T. H. Sabeh F. Ota I. Murphy H. McDonagh K. T. Holmbeck K. (2004). MT1-MMP-dependent neovessel formation within the confines of the three-dimensional extracellular matrix. *J. Cell Biol.* 167 757–767. 10.1083/jcb.200405001 15545316 PMC2172577

[B9] Crosby C. O. Zoldan J. (2019). Mimicking the physical cues of the ECM in angiogenic biomaterials. *Regen. Biomater.* 6 61–73. 10.1093/rb/rbz003 30967961 PMC6447000

[B10] Cross V. L. Zheng Y. Choi N. W. Verbridge S. S. Sutermaster S. A. Bonassar L. J. (2010). Dense type I collagen matrices that support cellular remodeling and microfabrication for studies of tumor angiogenesis and vasculogenesis in vitro. *Biomaterials* 31 8596–8607. 10.1016/j.biomaterials.2010.07.072 20727585 PMC2949514

[B11] Denais C. M. Gilbert R. M. Isermann P. McGregor A. L. te Lindert M. Weigelin B. (2016). Nuclear envelope rupture and repair during cancer cell migration. *Science* 352 353–358. 10.1126/science.aad7297 27013428 PMC4833568

[B12] Dogterom M. Koenderink G. H. (2019). Actin–microtubule crosstalk in cell biology. *Nat. Rev. Mol. Cell Biol.* 20 38–54. 10.1038/s41580-018-0067-1 30323238

[B13] Doyle A. D. (2016). Generation of 3D collagen gels with controlled diverse architectures. *Curr. Protoc. Cell Biol.* 72 10–20. 10.1002/cpcb.9 27580704 PMC5030718

[B14] Du Y. Herath S. Wang Q.-G. Wang D. A. Asada H. H. Chen P. C. Y. (2016). Three-dimensional characterization of mechanical interactions between endothelial cells and extracellular matrix during angiogenic sprouting. *Sci. Rep.* 6:21362. 10.1038/srep21362 26903154 PMC4763258

[B15] Francavilla C. Maddaluno L. Cavallaro U. (2009). The functional role of cell adhesion molecules in tumor angiogenesis. *Semin. Cancer Biol.* 19 298–309. 10.1016/j.semcancer.2009.05.004 19482088

[B16] Friedl P. Wolf K. (2009). Proteolytic interstitial cell migration: a five-step process. *Cancer Metastasis Rev.* 28 129–135. 10.1007/s10555-008-9174-3 19153672

[B17] Gerhardt H. Golding M. Fruttiger M. Ruhrberg C. Lundkvist A. Abramsson A. (2003). VEGF guides angiogenic sprouting utilizing endothelial tip cell filopodia. *J. Cell Biol.* 161 1163–1177. 10.1083/jcb.200302047 12810700 PMC2172999

[B18] Grashoff C. Hoffman B. D. Brenner M. D. Zhou R. Parsons M. Yang M. T. (2010). Measuring mechanical tension across vinculin reveals regulation of focal adhesion dynamics. *Nature* 466 263–266. 10.1038/nature09198 20613844 PMC2901888

[B19] Griffin D. R. Weaver W. M. Scumpia P. O. Di Carlo D. Segura T. (2015). Accelerated wound healing by injectable microporous gel scaffolds assembled from annealed building blocks. *Nat. Mater.* 14 737–744. 10.1038/nmat4294 26030305 PMC4615579

[B20] Hall M. S. Alisafaei F. Ban E. Feng X. Hui C.-Y. Shenoy V. B. (2016). Fibrous nonlinear elasticity enables positive mechanical feedback between cells and extracellular matrices. *Proc. Natl. Acad. Sci. U.S.A.* 113 1–46. 10.1073/pnas.1613058113 27872289 PMC5150395

[B21] Ham T. R. Collins K. L. Hoffman B. D. (2019). Molecular tension sensors: moving beyond force. *Curr. Opin. Biomed. Eng.* 12 83–94. 10.1016/j.cobme.2019.10.003 32864525 PMC7450811

[B22] Huxley V. Rumbaut R. (2000). The microvasculature as a dynamic regulator of volume and solute exchange. *Clin. Exp. Pharmacol. Physiol.* 27 847–854. 10.1046/j.1440-1681.2000.03344.x 11022981

[B23] Ilina O. Gritsenko P. G. Syga S. Lippoldt J. La Porta C. A. M. Chepizhko O. (2020). Cell–cell adhesion and 3D matrix confinement determine jamming transitions in breast cancer invasion. *Nat. Cell Biol.* 22 1103–1115. 10.1038/s41556-020-0552-6 32839548 PMC7502685

[B24] Juliar B. A. Keating M. T. Kong Y. P. Botvinick E. L. Putnam A. J. (2018). Sprouting angiogenesis induces significant mechanical heterogeneities and ECM stiffening across length scales in fibrin hydrogels. *Biomaterials* 162 99–108. 10.1016/j.biomaterials.2018.02.012 29438884 PMC5831523

[B25] Khetan S. Guvendiren M. Legant W. Cohen D. M. Chen C. S. Burdick J. A. (2013). Degradation-mediated cellular traction directs stem cell fate in covalently crosslinked three-dimensional hydrogels. *Nat. Mater.* 12 458–465. 10.1038/nmat3586 23524375 PMC3633615

[B26] Kinstlinger I. S. Miller J. S. (2016). 3D-printed fluidic networks as vasculature for engineered tissue. *Lab Chip* 16 2025–2043. 10.1039/C6LC00193A 27173478

[B27] Li L. Eyckmans J. Chen C. S. (2017). Designer biomaterials for mechanobiology. *Nat. Mater.* 16 1164–1168. 10.1038/nmat5049 29170549 PMC7001850

[B28] Loebel C. Mauck R. L. Burdick J. (2019). Local nascent protein deposition and remodeling guide mesenchymal stromal cell mechanosensing and fate in three-dimensional hydrogels. *Nat. Mater.* 18 883–891. 10.1038/s41563-019-0307-6 30886401 PMC6650309

[B29] Matera D. L. DiLillo K. M. Smith M. R. Davidson C. D. Parikh R. Said M. (2020). Microengineered 3D pulmonary interstitial mimetics highlight a critical role for matrix degradation in myofibroblast differentiation. *Sci. Adv.* 6:eabb5069. 10.1126/sciadv.abb5069 32917680 PMC11206459

[B30] Matera D. L. Wang W. Y. Smith M. R. Shikanov A. Baker B. M. (2019). Fiber density modulates cell spreading in 3D interstitial matrix mimetics. *ACS Biomater. Sci. Eng.* 5 2965–2975. 10.1021/acsbiomaterials.9b00141 33405599

[B31] Mirabella T. MacArthur J. W. Cheng D. Ozaki C. K. Woo Y. J. Yang M. T. (2017). 3D-printed vascular networks direct therapeutic angiogenesis in ischaemia. *Nat. Biomed. Eng.* 1:0083. 10.1038/s41551-017-0083 29515935 PMC5837070

[B32] Nguyen D.-H. T. Stapleton S. C. Yang M. T. Cha S. S. Choi C. K. Galie P. A. (2013). Biomimetic model to reconstitute angiogenic sprouting morphogenesis in vitro. *Proc. Natl. Acad. Sci. U.S.A.* 110 6712–6717. 10.1073/pnas.1221526110 23569284 PMC3637738

[B33] Novosel E. C. Kleinhans C. Kluger P. J. (2011). Vascularization is the key challenge in tissue engineering. *Adv. Drug Deliv. Rev.* 63 300–311. 10.1016/j.addr.2011.03.004 21396416

[B34] Osaki T. Kakegawa T. Kageyama T. Enomoto J. Nittami T. Fukuda J. (2015). Acceleration of vascular sprouting from fabricated perfusable vascular-like structures. *PLoS One* 10:e0123735. 10.1371/journal.pone.0123735 25860890 PMC4393106

[B35] Paik J. H. Chae Ss Lee M. J. Thangada S. Hla T. (2001). Sphingosine 1-phosphate-induced endothelial cell migration requires the expression of EDG-1 and EDG-3 receptors and Rho-dependent activation of alpha vbeta3- and beta1-containing integrins. *J. Biol. Chem.* 276 11830–11837. 10.1074/jbc.M009422200 11150298

[B36] Potente M. Gerhardt H. Carmeliet P. (2011). Basic and therapeutic aspects of angiogenesis. *Cell* 146 873–887. 10.1016/j.cell.2011.08.039 21925313

[B37] Rhee S. Jiang H. Ho C. H. Grinnell F. (2007). Microtubule function in fibroblast spreading is modulated according to the tension state of cell-matrix interactions. *Proc. Natl. Acad. Sci. U.S.A.* 104 5425–5430. 10.1073/pnas.0608030104 17369366 PMC1838480

[B38] Shakiba D. Alisafaei F. Savadipour A. Rowe R. A. Liu Z. Pryse K. M. (2020). The balance between actomyosin contractility and microtubule polymerization regulates hierarchical protrusions that govern efficient fibroblast-collagen interactions. *ACS Nano* 14 7868–7879. 10.1021/acsnano.9b09941 32286054

[B39] Shin D. S. Tokuda E. Y. Leight J. L. Miksch C. E. Brown T. E. Anseth K. S. (2018). Synthesis of microgel sensors for spatial and temporal monitoring of protease activity. *ACS Biomater. Sci. Eng.* 4 378–387. 10.1021/acsbiomaterials.7b00017 29527570 PMC5842818

[B40] Thomson K. S. Korte F. S. Giachelli C. M. Ratner B. D. Regnier M. Scatena M. (2013). Prevascularized microtemplated fibrin scaffolds for cardiac tissue engineering applications. *Tissue Eng. Part A* 19 967–977. 10.1089/ten.tea.2012.0286 23317311 PMC3589898

[B41] Traore M. A. George S. C. (2017). Tissue engineering the vascular tree. *Tissue Eng. Part B Rev.* 23 505–514. 10.1089/ten.teb.2017.0010 28799844 PMC5729878

[B42] Trappmann B. Baker B. M. Polacheck W. J. Choi C. K. Burdick J. A. Chen C. S. (2017). Matrix degradability controls multicellularity of 3D cell migration. *Nat. Commun.* 8:371. 10.1038/s41467-017-00418-6 28851858 PMC5575316

[B43] Vaeyens M. M. Jorge-Peñas A. Barrasa-Fano J. Steuwe C. Heck T. Carmeliet P. (2020). Matrix deformations around angiogenic sprouts correlate to sprout dynamics and suggest pulling activity. *Angiogenesis* 23 315–324. 10.1007/s10456-020-09708-y 31997048

[B44] van Helvert S. Storm C. Friedl P. (2018). Mechanoreciprocity in cell migration. *Nat. Cell Biol.* 20 8–20. 10.1038/s41556-017-0012-0 29269951 PMC5943039

[B45] Van Hinsbergh V. W. M. Koolwijk P. (2008). Endothelial sprouting and angiogenesis: matrix metalloproteinases in the lead. *Cardiovasc. Res.* 78 203–212. 10.1093/cvr/cvm102 18079100

[B46] Vigen M. Ceccarelli J. Putnam A. J. (2014). Protease-sensitive PEG hydrogels regulate vascularization in vitro and in vivo. *Macromol. Biosci.* 14 1368–1379. 10.1002/mabi.201400161 24943402 PMC4198447

[B47] Wang W. Y. Davidson C. D. Lin D. Baker B. M. (2019). Actomyosin contractility-dependent matrix stretch and recoil induces rapid cell migration. *Nat. Commun.* 10:1186. 10.1038/s41467-019-09121-0 30862791 PMC6414652

[B48] Wang W. Y. Lin D. Jarman E. H. Polacheck W. J. Baker B. M. (2020). Functional angiogenesis requires microenvironmental cues balancing endothelial cell migration and proliferation. *Lab Chip* 20 1153–1166. 10.1039/C9LC01170F 32100769 PMC7328820

[B49] Wei Z. Schnellmann R. Pruitt H. C. Gerecht S. (2020). Hydrogel network dynamics regulate vascular morphogenesis. *Cell Stem Cell* 27 798–812. 10.1016/j.stem.2020.08.005 32931729 PMC7655724

[B50] Wisdom K. M. Adebowale K. Chang J. Lee J. Y. Nam S. Desai R. (2018). Matrix mechanical plasticity regulates cancer cell migration through confining microenvironments. *Nat. Commun.* 9:4144. 10.1038/s41467-018-06641-z 30297715 PMC6175826

[B51] Wolf K. Friedl P. (2011). Extracellular matrix determinants of proteolytic and non-proteolytic cell migration. *Trends Cell Biol.* 21 736–744. 10.1016/j.tcb.2011.09.006 22036198

[B52] Wood L. B. Ge R. Kamm R. D. Asada H. H. (2012). Nascent vessel elongation rate is inversely related to diameter in in vitro angiogenesis. *Integr. Biol. (Camb).* 4 1081–1089. 10.1039/c2ib20054f 22847074

[B53] Yamada K. M. Sixt M. (2019). Mechanisms of 3D cell migration. *Nat. Rev. Mol. Cell Biol.* 20 738–752. 10.1038/s41580-019-0172-9 31582855

[B54] Yoon C. Choi C. Stapleton S. Mirabella T. Howes C. Dong L. (2019). Myosin IIA–mediated forces regulate multicellular integrity during vascular sprouting. *Mol. Biol. Cell* 30 1974–1984. 10.1091/mbc.E19-02-0076 31318321 PMC6727772

